# Differential coping strategies exerted by biofilm and planktonic cells of *Bacillus subtilis* in response to a protozoan predator

**DOI:** 10.1128/spectrum.01597-25

**Published:** 2025-11-14

**Authors:** Ilana Kolodkin-Gal, Prem Anand Murugan, Smruti Mahapatra, Eva Zanditenas, Serge Ankri

**Affiliations:** 1 Scojen Institute for Synthetic Biology, Dina Recanati School of Medicine, Reichman University 837206, Herzliya, Israel; 2 Department of Molecular Microbiology, Ruth and Bruce Rappaport Faculty of Medicine, Technion, Haifa, Israel; Rowan University Cooper Medical School, Camden, New Jersey, USA

**Keywords:** biofilms, *Entamoeba histolytica*, stress adaptation, stress response

## Abstract

The human protozoan parasite *Entamoeba histolytica* causes amebiasis and interacts with both beneficial and harmful members of the microbiome. In previous studies, it was shown that *E. histolytica* can break down pre-established biofilms of *Bacillus subtilis* in a time- and dose-dependent manner. Inhibiting parasitic cysteine proteases impairs biofilm degradation. However, it is still unknown whether bacteria can sense this process and respond to the degradation of the biofilms. Here, our research demonstrates a multilayered response of probiotic bacteria to the parasite, which differs between planktonic bacteria and pre-established biofilms. Sensing the activity of cysteine proteases from *E. histolytica*, the bacteria activate the general stress response and, to a lesser extent, the cell wall stress response. This activation helps the surviving members of the biofilm become more resistant to mild stressors such as ethanol, hydrogen peroxide, and sub-mic concentrations of ampicillin. On the other hand, planktonic cells exposed to the predators’ lysate deactivate the expression of genes associated with biofilm formation while inducing their motility to avoid predation. Overall, our results indicate that bacteria have evolved to recognize amoeba predators capable of degrading biofilms. Furthermore, the partially digested biofilm cells may have unexpected disadvantages over bacteria that did not encounter a predator. These findings may be useful in developing more efficient probiotic strains that are resilient to amebiasis.

**IMPORTANCE:**

The human protozoan parasite *Entamoeba histolytica* feeds on intestinal microbiota to survive. To enhance the effectiveness of probiotics, we characterized how they respond to amoeba predators. We found that probiotics decrease the expression of biofilm-related genes to avoid predation while simultaneously inducing their stress response and increasing their motility. Our results can provide novel directions for engineering probiotic bacteria to overcome gastrointestinal-associated parasitic diseases. Additionally, it highlights a fundamental mechanism through which bacterial prey can evade predation in the gastrointestinal tract.

## OBSERVATION

*Entamoeba histolytica* is a protozoan parasite responsible for amebiasis, a widely prevalent human intestinal disease in developing countries. Transmission of amebiasis occurs mainly through contaminated food or water with feces containing *E. histolytica* cysts, which is one of two forms of the parasite (1). On entering the host intestine, the cyst, the resistant form of the parasite, undergoes a process called excystation during which trophozoites, the vegetative form of the parasite, are released (2). In most cases of infection, these trophozoites feed on bacterial microbiota or cellular debris in the large intestine without causing symptoms.

*Bacillus subtilis* is a bacterium capable of forming biofilms. It is commonly found in soil and is used as a probiotic to support digestive and immune health in both adults and children. When grown in isolation, it forms robust biofilms both on liquid surfaces and agar plates, which are triggered by factors like low oxygen and nutrient depletion. The process is governed by the master regulator Spo0A. The initial step involves the transition of planktonic cells to a sessile state, with a decrease in flagella gene expression and an increase in genes responsible for producing the extracellular matrix (3, 4). This matrix consists of extracellular polysaccharides and proteins, with the proteins TasA and TapA contributing to the 3D architecture of the biofilms (5, 6), and these proteins are co-regulated with an additional protein barrier, the hydrophobin BslA (7). TasA regulates gene expression in the complex biofilm structures (8, 9) and promotes the formation of crystalline calcium carbonate (10).

In addition to biofilm formation, the transcriptional stress response in *B. subtilis* can be activated by the general stress response regulators, primarily the alternative sigma factor SigB (11). The activation of this sigma factor, transiently induced after the imposition of stress, allows *B. subtilis* to exhibit an immediate response to various stresses through the induction of over 150 general stress proteins (11).

Our previous research explored the molecular mechanisms involved in the interaction between parasites and biofilms that co-exist in the same habitat, specifically the gut (12). We used genetically manipulable models of *E. histolytica* and *B. subtilis* for our study. Once bacteria form biofilms, simple phagocytosis becomes unfeasible, as the biofilms grow too large to be engulfed. Therefore, the degradation of the biofilms and release of smaller particles are essential for the amoeba to feed (13). We identified the unique transcriptome of *E. histolytica* after exposure to *B. subtilis* biofilm, the role of cysteine proteases in the degradation of the biofilm, and the protective role of the *B. subtilis* biofilm against oxidative stress. These results highlighted the differential response of the parasites to planktonic and biofilm cells and the co-evolution of *E. histolytica* parasites and biofilms (12). However, the response of the bacteria to the parasite and whether it is different between planktonic cells and biofilm cells remain to be determined.

In this work, we deepen our understanding of the response of biofilm cells to the predator and the predation process as judged by their response to active cysteine proteinases (CPs) from the extract. Following predation, biofilm cells lose their tolerance to stressors due to the dissolution of the extracellular matrix. Our results indicate that biofilm cells non-specifically sense CPs and activate the general stress response genes. In addition, biofilm cells activate β-lactamase associated with the cell wall stress response, which allows them to survive moderate stressors and antibiotic concentrations upon release to the environment. During liver infection, a massive death of trophozoites is observed in the first hours post-infection due to the activation of the immune response, releasing lysate content into the bloodstream and subsequently to the gastrointestinal tract (14). Death of free-living trophozoites may also occur during cell division and with exposure to disinfectants (15).

Hence, exposure to the parasite lysate may act as a warning signal for bacteria, prompting them to avoid forming biofilms and instead promoting movement. This ability enables them to effectively evade predation by moving away from potential predators. Interestingly, the stress response is activated in established biofilms, while the downregulation of matrix genes is more common in planktonic bacteria. Overall, our findings suggest that biofilm-forming cells have co-evolved with potential predators, enabling prey to survive and co-exist in the same environment as their predators.

### Culture of *E. histolytica*

*E. histolytica* trophozoites, the HM-1:IMSS strain (from Prof. Samudrala Gourinath, Jawaharlal Nehru University, New Delhi, India), were grown and harvested according to a previously reported protocol (16).

### Extract preparation

*E. histolytica* trophozoites (1 × 10^6^) total lysates were prepared using a lysis buffer containing 1% Nonidet P-40 (NP-40 in deuterium-depleted water. The trophozoites were incubated for 15 min with NP-40 on ice and centrifuged for 10 min at 12,000 rpm at 4°C. The supernatant was then transferred to a new tube. Protein concentration was measured by Bradford. For boiled aliquots, the lysate was boiled at 100°C for 5 min. Complete protein denaturation was verified by remeasuring protein concentration in the boiled extracts.

### Strains and media

All strains were derived from *B. subtilis* NCIB 3610 (17). The strains were routinely grown in Luria-Bertani (LB) broth (Difco) or MSgg medium (17) (5 mM potassium phosphate, 100 mM MOPS [pH 7], 2 mM MgCl_2_, 50 µM MnCl_2_, 50 µM [liquid assay] and 125 µM [biofilm assay] FeCl_3_, 700 µM CaCl_2_, 1 µM ZnCl_2_, 2 µM thiamine, 0.5% glycerol, 0.5% glutamate, 50  µg/mL threonine, tryptophan, and phenylalanine). Solid medium contained 1.5% Bacto agar (Difco). Papain solution was prepared with P3375, Sigma-Aldrich papain from papaya latex (crude powder, 1.5–10.0 units/mg).

### Colony preparation and extract analysis

Colonies were grown on MSgg medium and incubated at 30°C as described by us previously (18, 19). To determine the effect of the amoeba’s lysate, the 48 hour biofilms were harvested. Colonies were incubated in MSgg containing the indicated concentrations of phosphate-buffered saline (PBS)/indicated concentrations of extract/indicated concentrations of extract + E64D for 4 hours/boiled extracts/papain solution. The treated colonies were incubated in 30°C. Extracts were diluted vol/vol from a stock of 2 ng/µL.

#### Response to stressors

**Fig 1:**
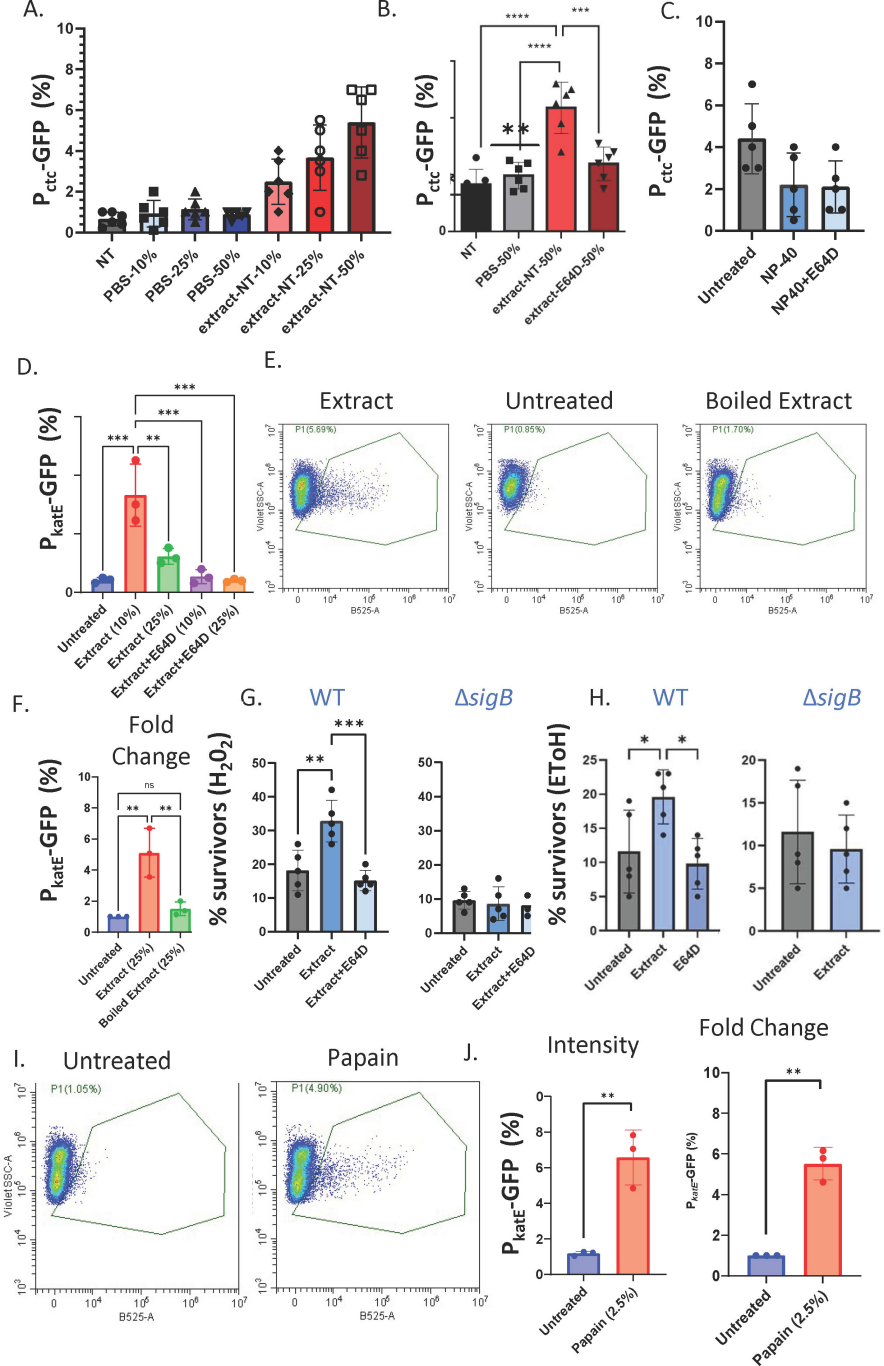
Amoeba CP activity triggers the bacterial stress response. (**A–C**) The green fluorescent protein (GFP) fluorescence from *B. subtilis* strain NCIB 3610 harboring *amyE::Pctc-GFP* (a reporter for the general stress response) was analyzed in the presence and absence of increasing concentrations of extracts or NP-40 lysis buffer control from *E. histolytica* using flow cytometry. The NT extract at concentration of 25% and 50% differed from the untreated control (**P < 0.01, ****P < 0.001). (**B**) Extracts with and without E64D were used at identical concentrations (diluted vol/vol from a stock of 2 ng/µL). (C) The 48 hour biofilms were resuspended in 100 µL of indicated solution representing 50% extract working concentrations (0.2% NP-40, with/without E64D 5 µM). From untreated and treated biofilms, 100,000 cells were counted with flow cytometry. The percentage of cells expressing the reporters was calculated. Graphs represent mean ± SD from six independent experiments (*n* = 3). (**D**) The GFP fluorescence from *B. subtilis* strain NCIB 3610 harboring *amyE:PkatE-GFP* (a reporter for the general stress response and of the gene encoding for a general catalase) was analyzed in the presence and absence of increasing concentrations of extracts from *E. histolytica* using flow cytometry as indicated above. (**E and F**) *B. subtilis* strain NCIB 3610 harboring *amyE::PkatE-GFP* was analyzed in the presence and absence of extracts/boiled extracts (25%) from *E. histolytica* using flow cytometry. From untreated and treated biofilms, 100,000 cells were counted. The percentage of cells expressing the GFP reporter was calculated. Graphs represent SD from nine independent experiments (*n* = 3). (**G and H**) *B. subtilis* strain NCIB 3610 and its Δ*sigB* mutant derivative were grown on MSgg agar at 30°C for 48 hours. Then, the colonies were cut in half with a razor blade as in references 18 and 19. Both halves were resuspended in 100 µL PBS (untreated)/25% extract (Extract)/25% extract + E64D (Extract + E64D) for 4 hours. Cells from each group were pelleted. A pellet of half a colony of each colony was resuspended either in PBS or resuspended in 500 µL PBS or with (**G**) hydrogen peroxide (10 mM) and (**H**) 50% (vol/vol) ethanol. Biofilm cells were further incubated for 20 min, washed, and plated to assess the percentage of survivors for each treatment. The percentage of surviving CFU is represented by the ratio of biofilm cells treated by the sterilizing agents compared with the same untreated group either exposed or unexposed to the extract. Data represent the average and SD of five independent experiments performed in duplicate. *P < 0.05, **P < 0.01, ***P < 0.001. (**I and J**) Papain was used at 2.5% concentration (diluted vol/vol from a stock of 10 mg/mL). Flow cytometry was performed as in panel **G**. ns, non-signficant was designated as *P* > 0.05.

To determine the susceptibility to ethanol or peroxide within a biofilm, the cell-number percentage of CFU without or with chemical stress was compared. Therefore, biofilms were grown on solid MSgg medium (18, 19). After 48 hours, the colonies were cut in half with a razor blade and separated into different microcentrifuge tubes, yielding an average of 10% error rate due to minor colony asymmetry (18, 19). First, both halves of each colony were treated in either PBS or extract or extract + E64D as described in the legend of Fig. 1G and H. Then biofilms were centrifuged (5 min at 14,000 rpm).

The pellet from one-half of the colony was exposed to a stressor as indicated. The pellet from the second half of the colony was incubated in PBS. Following incubation with the stressors, the supernatant was removed, and biofilms were resuspended in 500 µL PBS and mildly sonicated (amplitude 20%, pulse 3 × 5 s). The number of CFUs was determined by plating serial dilutions on LB plates and counting colonies after incubation at 30°C overnight. The percentage of surviving CFU is represented by the ratio of biofilm cells treated by the sterilizing agents compared to untreated (PBS) cells or to untreated (PBS) cells for the controls.

### Flow cytometry

Treated colonies were processed by sonication using a BRANSON digital sonicator. After sonication, samples were diluted in PBS and measured using an LSR‐II cytometer (Becton Dickinson, San Jose, CA, USA). The GFP fluorescence was measured using laser excitation of 488 nm, coupled with 505 LP and 525/50 sequential filters. To distinguish background fluorescence from the reporters’ specific fluorescence, the wild-type *B. subtilis* grown under the same conditions was used as a negative control, and its background fluorescence was gated to separate the true fluorescent population (population outside the background gate) from the reporters, as done in Maan et al. (20). A total of 100,000 cells were counted for each sample, and flow cytometry analyses were performed using FACS Diva (BD Biosciences) and FCS Express 7 Research Edition.

For Fig. 1D, E, I, and J, flow cytometry was performed on a CytoFLEX S with manufacturer-default fluorescence detector setup for violet laser (V450, 450/45 nm, V525, 525/40) and data acquisition with CytExpert software, including recording 100,000 events with a flow rate of ~3,500 events per second. Quality control was performed using fluorescent beads as per the manufacturer’s protocol. For the negative control, a sample with no fluorescence was used. Extended cleaning cycles were performed manually using Flow Clean reagent supplied by the manufacturer and double-distilled water at the beginning and end of each flow cytometry session, verifying a low event rate in filtered PBS.

### Statistics

All experiments were analyzed by GraphPad software (license to the Scojen Institute) with analysis of variance test for multiple comparisons, unless stated otherwise.

### *B. subtilis* biofilm cells induce the general stress response following exposure to CPS

We previously demonstrated that *B. subtilis* biofilm cells serve as prey for *E. histolytica* trophozoites (12). This gradual degradation of the established biofilms could trigger alterations in gene expression (4). Specifically, it was shown that the protein TasA, degraded by the parasite, is a regulator of the general stress response (8). Therefore, we evaluated the general stress response of the bacterium following exposure to amoeba extract. In short, we exposed pre-established biofilm cells to the extract for 30 min in increased doses of the extract and then evaluated their transcriptional response. To specifically monitor the general stress response, we assessed the transcription from the *ctc* promoter, directly regulated by SigB (21) in biofilm cells before and following exposure to the amoeba signal. Our results demonstrated a significant dose-dependent induction of the *ctc* promoter (Fig. 1A).

We then asked whether this induction depends on the degradation of biofilms by amoeba CPs (Fig. 1A). We utilized the cysteine proteinase inhibitor E64D, which allows for the expression of the CPs by the parasite but prevents their activity and consequently biofilm and *tasA* degradation (12). Our results indicated that the extract with the chemical inhibitor for CP activity E64D failed to induce the activation of SigB-dependent transcription (Fig. 1B). This result suggests that the stress response is a readout of the biofilm degradation activity of the CPs rather than their mere presence.

The extract and extraction buffer were not toxic to pre-established biofilm cells at the tested concentrations (Fig. S1). The buffer and E64D showed some inhibition of transcription from the *ctc* promoter (Fig. 1C), which was significantly less than the pronounced induction of the activity from the promoter treated with extract. Thus, the activation of the stress response is specific and serves as a physiological indicator of biofilm degradation. To further confirm the specific response of SigB-dependent promoters to *B. subtilis* biofilm degradation by amoeba CPs, we tested the transcription from an additional SigB-dependent promoter, *katE* promoter (22) (encoding a catalase). This promoter is almost exclusively under SigB control in non-sporulation conditions. As shown, the exposure of biofilm cells to amoeba extract induced the transcription from the *katE* promoter and was dependent on the presence of CPs (Fig. 1D). Moreover, the *katE* promoter did not respond to the presence of boiled *E. hystolytica* extract (Fig. 1E and F), strongly indicating the response is a response to the degradation of the biofilm. These results confirm that exposure to amoeba extract and subsequent biofilm degradation by CPs activate the general stress response in biofilm cells.

It was previously demonstrated that the *SigB* mutant impairs growth under ethanol and hydroxyl radical stress but does not affect growth under normal conditions. Therefore, we suspected that the induced stress response contributed to the increased tolerance to hydroxyl radicals. Consistently, we found that the pre-established biofilm cells treated with amoeba extract were indeed more resistant to H_2_O_2_ (Fig. 1G), and this was dependent on the presence of the amoeba extract and active CPs. Similar results were obtained with EtOH (Fig. 1H). Therefore, the activation of the general stress response during biofilm degradation is associated with elevated stress tolerance for the mild general stressors studied here. These results are supported by the activation of the *katE* promoter by commercial papain (Fig. 1I and J), a non-specific endopeptidase from papaya latex with wide substrate range and esterase and thiol-esterase activities.

### *B. subtilis* biofilm cells induce the cell wall stress response in response to predation

The protein TasA, targeted by CPs from *E. histolytica* for degradation, is in an intimate connection with the cell envelope, and its degradation in pre-established biofilms may disturb the integrity of matrix-associated cell envelopes. Therefore, we tested whether cells from the partially degraded biofilms experience elevated stress following their exposure to the predator. Four sigma factors—SigM, SigW, SigX, and SigV—play crucial roles in maintaining cell envelope homeostasis, with the SigW mutant having the most robust phenotype as a single mutant (23).

**Fig 2:**
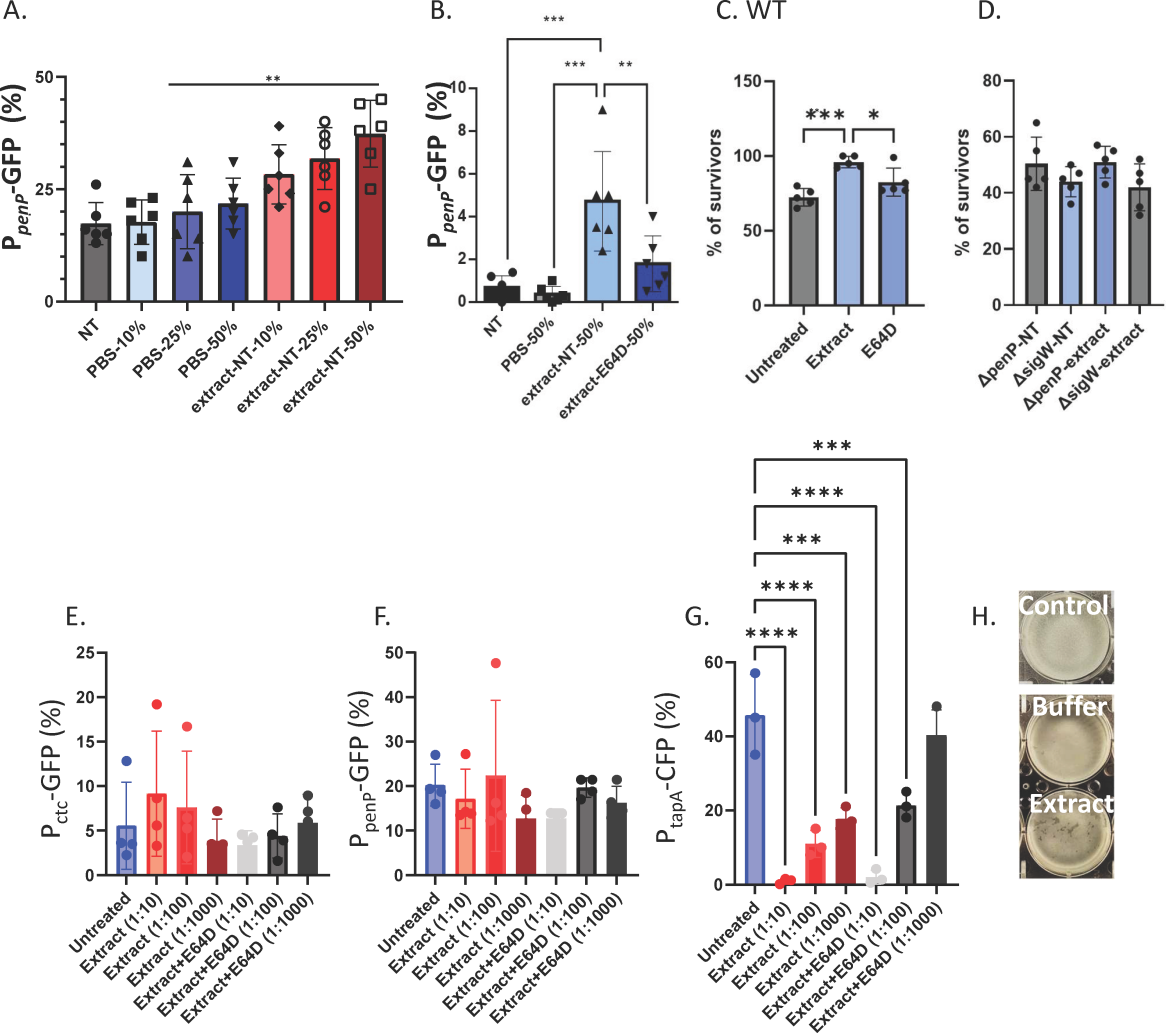
The adaptation of biofilm cells and planktonic cells to amoeba CPs. (**A**) *B. subtilis* NCIB 3610 strain harboring *amyE::PpenP-GFP* (cell wall stress response) was analyzed in the presence and absence of increasing concentrations of extracts from *E. histolytica* using flow cytometry. Colonies were grown on MSgg medium and incubated at 30°C. To determine the effect of amoeba’s lysate, the 48 hour biofilms were harvested. Colonies were incubated in indicated concentrations of PBS/indicated concentrations of extract/indicated concentrations of extract + E64D for 4 hours. Extracts were diluted vol/vol from a stock of 2 ng/µL. From untreated and treated biofilms, 100,000 cells were counted. The percentage of cells expressing the reporters was calculated. Graphs represent mean ± SD from six independent experiments (*n* = 3). (B) Extracts with and without E64D were used at identical concentrations (diluted vol/vol from a stock of 2 ng/µL). (**B**) As in panel A, except the colonies were resuspended in 100 µL PBS/extract or PBS/extract + E64D for 4 hours. Extracts were diluted vol/vol from a stock of 2 ng/µL. **P < 0.01, ***P < 0.001. (**C and D**) *B. subtilis* NCIB 3610 wild-type (WT) (**C**) and its indicated mutants (**D**) were grown on MSgg agar at 30°C for 48 hours. Then, the colonies were scrapped from the agar and resuspended in 100 µL PBS/extract/extract + E64D for 4 hours. Extracts were diluted vol/vol from an identical stock of 2 ng/µL in lysis buffer. Cells were pelleted and resuspended in 500 µL PBS with ampicillin (60 ng/mL) for 4 hours. Following incubation, biofilm cells were centrifuged (5 min at 14,000 rpm); the supernatant was removed; and biofilms were resuspended in 500 µL PBS and mildly sonicated (amplitude 20%, pulse 3 × 5 s). The number of CFUs was determined by plating serial dilutions on LB plates and counting colonies after incubation at 30°C overnight. The percentage of surviving CFUs is represented by the ratio of biofilm cells treated by ampicillin to their untreated counterpart. Data represent the average and SD of five independent experiments performed in duplicate. *P < 0.05, ***P < 0.001. (**E–G**) Planktonic response to extract: *B. subtilis* cells carrying each of the indicative reporters were grown logarithmically with shaking. At OD = 0.6, cells were pelleted and resuspended in 100 µL in MSgg containing the indicated concentrations of PBS/indicated concentrations of extract/indicated concentrations of extract + E64D for 4 hours. A total of 100,000 cells were counted with flow cytometry from untreated and planktonic cells. The percentage of cells expressing the reporters was calculated. Graphs represent SD from four independent experiments performed in technical duplicate. **P < 0.01, ***P < 0.001, ****P < 0.0001. (**H**) Pellicle formation was assessed following 18 hours of growth in MSgg medium at 30°C either untreated (control) or with 0.1% buffer/extract as indicated.

Our previous results demonstrated that SigW plays a central role in the activation of the β-lactamase PenP (24) to provide resistance to nanomolar concentrations of β-lactam antibiotics (25). The exposure of pre-established biofilms to amoeba extracts significantly induced transcription from PenP promoter in a dose-dependent manner (Fig. 2A), although the level of induction (as judged by the difference between the extract treated group and the basal activity levels of the promoter) was milder than the induction of the general stress response (Fig. 2B). The activation depended on the proteolytic activity of the amoeba’s CPs (see Fig. 2B). The transcription from *penP* promoter was not affected by the buffer or the presence of E64D (see Fig. S2). These results suggested that disrupting *B. subtilis* biofilms, rather than the presence of the amoeba’s CP and extract components, enhances the general stress response (Fig. 1) and the cell wall stress response (Fig. 2A and B).

Our previous results showed that due to the complete degradation of treated *B. subtilis* biofilms, the biofilm cells became sensitive to the treatment of toxic concentrations of ampicillin (600 ng/mL ampicillin). The restoration of the response to ampicillin was a result of the breakdown of the extracellular matrix by CPs (12). However, PenP only provides resistance to ampicillin at low nanomolar concentrations (25).

Therefore, we researched if activating PenP could safeguard cells impacted by the parasite when exposed to lower concentrations of ampicillin (60 ng/mL ampicillin). Our findings indicated a mild PenP-mediated protection against ampicillin (Fig. 2C and D), which relied on the activation of SigW. The effect of the *E. histolytica* extract appeared to be partly reliant on CPs (see Fig. 2D). In general, exposure to extract from *E. histolytica* had a more significant impact on the overall stress response and subsequent tolerance to related antibacterial stressors (e.g., H_2_O_2_) compared to the response to cell wall stress.

### The exposure of planktonic cells to the extract reduces significantly the formation of biofilms

In planktonic cultures, the extract was toxic, in contrast to its lack of effect on biofilm cells. This toxicity was not dependent on the activity of CPs (Fig. S3). Consequently, we only assessed the impact of amoeba extract on expression in non-toxic conditions. Under these conditions, we did not observe any evidence of the activation of the SigB-dependent promoter katE and the promoter of SigW-dependent *penP* promoter by the lysate of *E. histolytica*. These results suggest that pre-established biofilm cells respond to amoeba differently from planktonic bacteria (Fig. 2E).

Since it is not advantageous to form a biofilm in the presence of an efficient predator, we investigated whether a lysate from an amoeba predator could alter the dynamics of biofilm formation. To address this, we examined how the amoeba extract influences the expression of biofilm genes. Our findings revealed that the transcription from the *tapA* promoter, which controls the expression of the *tapA-sipW-tasA* operon (26, 27), is significantly reduced in the presence of nanograms of amoeba extract (Fig. 2E). This result is consistent with the repression of biofilm formation with amoeba extract (Fig. 2G). Moreover, a similar repression was observed in the presence of E64D but not with the buffer (Fig. 2G; Fig. S4). Interestingly, the repression of the *tapA* promoter was not significant in treated biofilm cells (Fig. S5), further emphasizing a differential response of the biofilm cells and their planktonic counterparts.

### Conclusion

*B. subtilis* biofilms engage in predator-prey interactions with protist parasites that can degrade pre-established biofilms pre-established biofilms (12, 13). This degradation results from the specific activation of the amoeba’s CPs in the biofilm, which target and break down the biofilm’s protein matrix (12). The response of biofilm cells to changes in the microenvironment caused by parasites remains unclear. In this study, we demonstrate that the degradation of biofilms by amoeba CPs triggers a general stress response (Fig. 1). This response involves the activation of a broad array of stress proteins, which are induced by various forms of physical stress, including heat, salt, ethanol, and acid stress. As a result, this stress response may serve as a relatively non-specific yet crucial protective function in the face of stress, regardless of the specific stressor. In our study, we have shown that the genes targeted by SigB are activated by the activity of amoeba’s CPs. Our conclusion is supported by the fact that the stress response induced by CPs is reversed in the presence of E64D. When the chemical inhibitor E64D is used, the CPs are present but inactive. This suggests that the dissolution of the biofilm imposes significant stress on the envelope of *B. subtilis*, leading to the activation of the extra cytoplasmic sigma factor SigW (as shown in Fig. 2). It is worth noting that the stress signal is a result of the degradation of the biofilm rather than the presence of the CPs, as the transcriptional response was not observed with boiled extract and could be restored with commercial papain (Fig. 1). These findings suggest that bacteria have evolved to perceive biofilm degradation as a stressor rather than simply a specific dispersal cue.

Moreover, the differential response of bacterial cells at planktonic stage and biofilm stage is indicative of a fundamentally different defense strategy of the bacteria. As biofilm dissolves, the bacteria lose their physical and chemical protection by the extracellular matrix and require adaptation to potentially unsheltered environments. The coupling of the activation of genes involved in stress protection with a general cue for biofilm degradation is beneficial to allow the bacteria surviving predation to increase their fitness while migrating to a less endangered environment. Interestingly, the response of planktonic bacteria to *E. histolytica* lysate suggests that planktonic bacteria sense and avoid biofilm formation in the presence of amoeba predators. The signal may be of relevance to trophozoites’ lysis during their rapid replication or during exposure to antiparasitic drugs or may also be represented in their secretion or even volatile profile.

Our discovery that *B. subtilis* biofilm genes are repressed in the presence of the parasite suggests that biofilm predation by amoeba (12) selected for alternative evasion strategies. As bacteria detect the approaching predator, they inhibit the activation of biofilm genes responsible for the formation of a non-mobile community (8) near the parasite. Downregulation of biofilm formation allows the bacteria to migrate toward alternative environments. Interestingly, the differential responses of planktonic bacterial cells and biofilm cells indicate that the differentiation into a multicellular community also influences their responses to predators and aggressors. This response of *B. subtilis* to a protist predator may depend on the biofilm predation capacity of the amoeba, as recent work also demonstrated that *B. subtilis* biofilm formation can provide protection versus the soil-dwelling amoeba, *Dictyostelium discoideum* (28).

*B. subtilis* is a well-established model for studying interspecies interactions (29). However, it can also be found in the human gastrointestinal tract and has been widely used in traditional fermented foods in many East Asian cultures for centuries (30). Additionally, it is emerging as a probiotic that may promote digestive health and support a healthy immune system (31, 32). Therefore, while the use of spore-forming bacteria as probiotics in humans remains a topic of discussion (33), this work could be of relevance to human health. Moreover, collectively, our results suggest that the antagonistic co-evolution between biofilm prey and protist parasites significantly impacts the evolution of bacterial transcriptional regulation.

## SUPPLEMENTAL MATERIAL

Supplemental figures spectrum.01597-25-s0001.pdf Figures S1 to S6.

ASM does not own the copyrights to Supplemental Material that may be linked to, or accessed through, an article. The authors have granted ASM a non-exclusive, world-wide license to publish the Supplemental Material files. Please contact the corresponding author directly for reuse.
